# Trends and factors associated with the utilisation of antenatal care services during the Millennium Development Goals era in Tanzania

**DOI:** 10.1186/s41182-020-00226-7

**Published:** 2020-06-03

**Authors:** Abdon Gregory Rwabilimbo, Kedir Y. Ahmed, Andrew Page, Felix Akpojene Ogbo

**Affiliations:** 1 Translational Health Research Institute, School of Medicine, Western Sydney University, Campbelltown Campus, Campbelltown, NSW Australia; 2 College of Medicine and Health Sciences, Samara University, P O Box 132, Samara, Ethiopia; 3 General Practice Unit, Prescot Specialist Medical Centre, Makurdi, Benue State Nigeria

**Keywords:** Antenatal care, Maternal and child health, Tanzania

## Abstract

**Background:**

A detailed understanding of trends, as well as what act as enablers and/or barriers to the utilisation of antenatal care (ANC) among Tanzanian women, is essential to policymakers and health practitioners to guide maternal health efforts. We investigated the trends and factors associated with ANC service use during the Millennium Development Goals (MDG) era in Tanzania between 1999 and 2016.

**Methods:**

The study used the Tanzania Demographic and Health Survey (TDHS) data for the years 1999 (*n* = 2095), 2004–2005 (*n* = 5576), 2010 (*n* = 6903) and 2015–2016 (*n* = 5392). Multivariate multinomial logistic regression models were used to investigate the association between predisposing, enabling, need and community-level factors and frequency of ANC (1–3 and ≥ 4) visits in Tanzania.

**Results:**

The proportion of women who made one to three ANC visits improved significantly from 26.4% in 1999 to 47.0% in 2016. The percentage of women who make four or more ANC visits declined from 71.1% in 1999 to 51.0% in 2016. Higher maternal education, belonging to wealthier households, being informally employed and listening to the radio were associated with four or more ANC visits. Women who did not desire pregnancy had a lower likelihood to attend four or more ANC visits. Women who had primary or higher education, those who resided in wealthier households and those who were informally employed were more likely to make between one and three ANC visits.

**Conclusion:**

The study showed that there was an improvement in the proportion of Tanzanian women who made one to three ANC visits, but it also indicated a concurrent decrease in the prevalence of four or more ANC visits. Improving uptake of ANC among Tanzanian women is achievable if national health policies and programmes also focus on key amenable maternal factors of education, household wealth and employment.

## Background

ANC is an effective healthcare strategy to improve maternal and newborn health and survival during pregnancy and childbirth. In 2018, the WHO estimated that approximately 303,000 women die every year (equivalent to 830 maternal deaths each day) from preventable pregnancy- and/or childbirth-related causes worldwide [1]. More than 99% (302,000 deaths) of these deaths occurred in low- and middle-income countries (LMICs) [1, 2]. In these countries, sub-Saharan African countries (including Tanzania) accounted for a larger number of those deaths (202,000 deaths per year) [1, 3].

In LMICs, common causes of maternal mortality are classified into direct and indirect [2, 4]. Direct causes include haemorrhages, hypertensive disorders, eclampsia, sepsis, abortion complications and obstructed labour [5], while indirect causes include severe anaemia, human immunodeficiency virus/acquired immunodeficiency disease syndrome (HIV/AIDS) complications and severe malaria [6]. Comprehensive ANC from skilled medical personnel is the main intervention to identify, manage and/or prevent these causes of maternal deaths [7, 8]. Based on the benefits of ANC service use, the WHO recommends that every pregnant woman should receive at least four ANC visits, which is a proxy indicator for comprehensive ANC [9–11]. However, between 2007 and 2014, reports indicated that only 64% of pregnant women received the four or more ANC visits globally, and less than half (45.8%) of pregnant women from LMICs received four or more ANC visits [11, 12]. These reports suggest that many pregnant women in LMICs do not take up routine ANC, with possible adverse implications for both mother and baby [11].

Although Tanzania was one of the countries that achieved a key target of the Millennium Development Goals (MDG-4, to reduce child mortality) [13, 14], recent evidence from the country has indicated that 8200 maternal deaths occurred in 2015 [7]. This suggests that MDG-5 (to improve maternal health) still needs significant attention in Tanzania. A recent report also indicated that an estimated 51% of Tanzanian women received the recommended four or more ANC visits in 2016, with wide variations across regional areas of the country [15]. Additionally, a previous national study conducted in Tanzania indicated that higher maternal education was associated with four or more ANC visits. In contrast, long distances to health facilities, not residing in Eastern Tanzania, never being married and a desire to avoid pregnancy were associated with underutilization of ANC service [16]. Although useful, this previous study has several limitations which include (i) non-inclusion of the most recent national data (2015–2016 Tanzania Demographic and Health Survey, TDHS), which potentially reflects the current socio-economic, demographic, health and political situation of the country [17, 18]; (ii) a lack of assessment of whether the recommended ANC visits (≥ 4) has improved or worsened over time that covers the MDGs era (2000–2015); and (iii) a lack of assessment of trends and determinants of incomplete ANC attendance (1–3 visits), which can provide valuable insights into where specific and measurable maternal health interventions can be provided to reach the recommended ANC attendance rates. Some sub-national studies have also been conducted in Tanzania on ANC service use, but their findings are unlikely to inform nationwide policy formulation and advocacy [19–22].

A detailed understanding of how ANC service use among Tanzanian women may have changed over time, especially during the MDG period, and identifying determinants associated with any change in ANC service use would be helpful to healthcare practitioners and policymakers. This context-specific information will help to inform efforts in the United Nation’s Sustainable Development Goals [23] era, with SDG–3.1 aiming to reduce the global maternal mortality ratio to less than 70 per 100,000 live births by 2030 [24]. The study findings will also be useful to strategic policy interventions that aim to scale up and/or reassess current maternal health commitments to improve the uptake of ANC services in Tanzania. The present study aimed to investigate the trends and factors associated with ANC service use during the MDGs era in Tanzania between 1999 and 2016.

## Methods

### Data sources

The study used the TDHS data for 1999 (*n* = 2095), 2004–2005 (*n* = 5576), 2010 (*n* = 6903), and 2015–2016 (*n* = 5392). The TDHS collected information on maternal health (e.g. ANC, birth, and postnatal), child health, infant nutrition, and other health-related data from nationally representative populations in Tanzania [15, 25–27]. The TDHS was implemented by the National Bureau of Statistics, Office of the Chief Government Statistician (OCGS) in Zanzibar and Inner City Fund (ICF) International, with funding from the Government of Tanzania, Global Affairs Canada and the United States Agency for International Development [28].

The TDHS used a two-stage stratified cluster sampling technique to select the study participants. In stage one, enumeration areas (EAs) were selected proportional to each geographical zones of Tanzania. The EAs used were based on 1988 (1999 TDHS), 2002 (2004–2005 and 2010 TDHS) and 2012 (2015–2016 TDHS) Tanzania Population and Housing Censuses [29, 30]. In stage two, a systematic random sampling technique was used to select households after the complete household listing was conducted in each EAs. The present study included a total weighted sample of 20,062 women who were pregnant or had given live birth within 5 years before the survey, consistent with the TDHS reports [15, 25–27] and past studies [31–33], to reduce the potential effect of recall bias. The response rates in the surveys ranged from 96% in 1999 to 98% in 2015–2016. Detailed methodological approaches used in the surveys are provided in the respective TDHS reports [15, 25–27].

### Outcome variables

The outcome variable was the frequency of ANC visits measured based on maternal recall to ANC service received from a doctor, nurse/midwife or any health personnel trained in maternal and child health assessment and management [11]. Consistent with the WHO recommendation [11] and previously published studies from LMICs [32, 34], frequency of ANC visits was categorised as ‘No ANC visit’, ‘one to three ANC visits’ and ‘four or more ANC visits’. In the analyses, the no ANC visit group was the reference category of the outcome variables as used in previous studies from LMICs [35–39].

### Study factors influencing the utilization of ANC

**Fig. 1:**
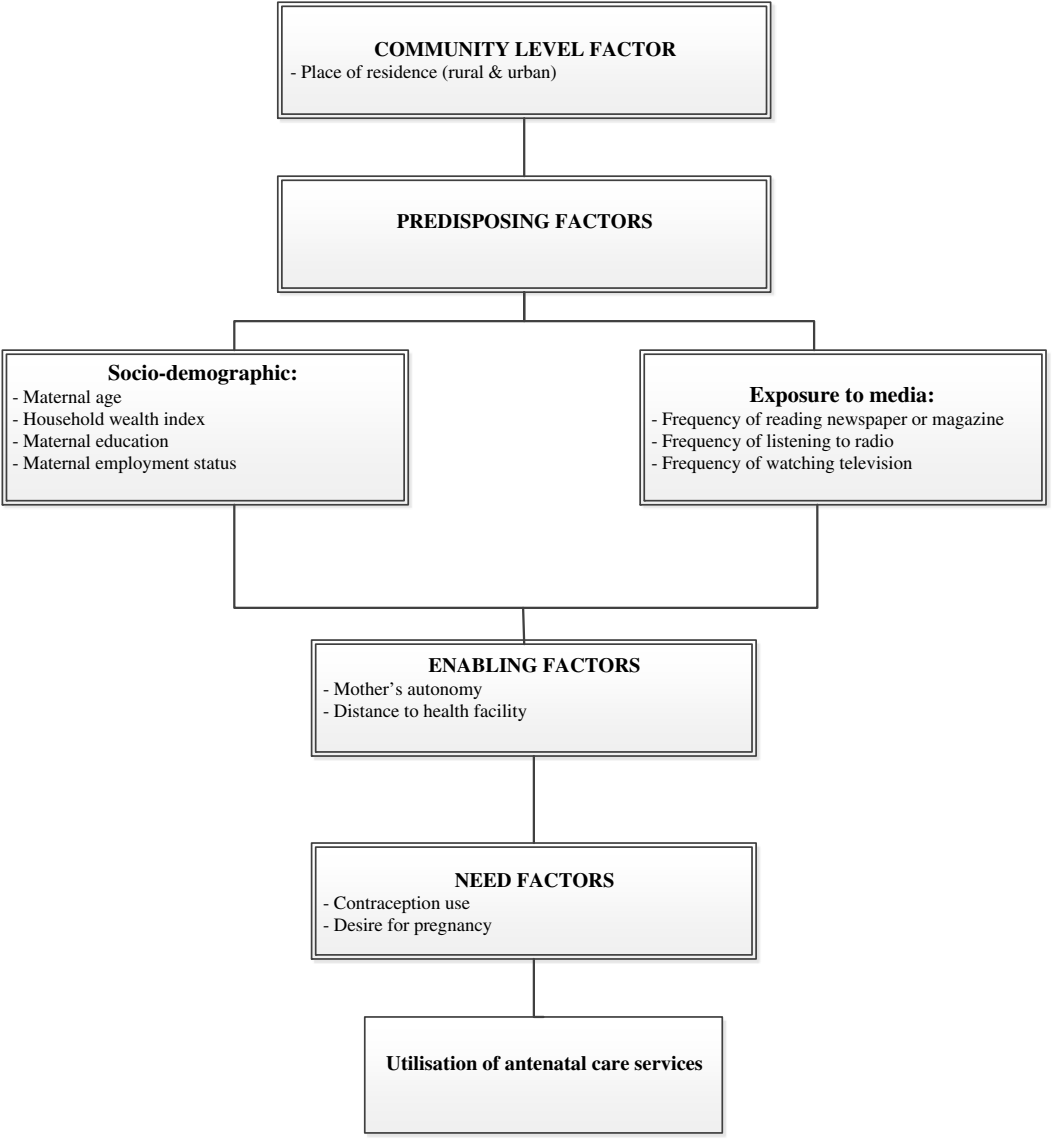
The conceptual model for ANC service use in Tanzania adapted from Anderson’s health service utilization model

Study factors were selected based on past studies from LMICs [14, 33, 34, 40, 41] and data availability in the TDHS. These factors were broadly classified into predisposing, enabling, need and community-level factors based on the conceptual model of health services utilization, proposed by Andersen to explain the conditions that favour or hinder an individual from seeking health care [42]. Our adapted conceptual approach was also consistent with past studies which had demonstrated relationships between the study factors and frequency of ANC service use [34, 37, 40, 43–45] (Fig. 1).

Community-level factors included the place of residence (categorised as rural or urban). Predisposing factors included socio-demographic factors and exposure to the media. Sociodemographic factors included maternal age (categorised as 15–24 years, 25–34 years or 35 and above years), mother’s education (categorised as no schooling or primary school and higher), mother’s employment (categorised as no employment, informal employment or formal employment) and household wealth index (categorised as poor, middle or rich). Informal employment included agricultural jobs, self-employed, skilled manual and unskilled manual works, while formal employment included professional work, technical, management, sales and clerical jobs. Household wealth index was computed by the National Bureau of Statistics and ICF using principal component analysis (PCA) which considered the ownership of household assets such as toilets, electricity, television, radio, fridge and bicycle, as well as availability of source of drinking water and floor material of the main house [46]. The TDHS categorized the household wealth index into lowest, second, middle, fourth and highest. For this study, the household wealth index was re-classified as ‘poor’, ‘middle’ or ‘rich’, consistent with previously published studies [47–49]. In those studies, the bottom 40% of the households were referred to as the poor households, the next 40% as the middle-level households and the top 20% as the rich households. In this study, maternal education and household wealth index were re-categorised for consistency and comparison with similar published studies conducted in LMICs [31, 32, 34], and to increase the statistical power within each sub-category of the variables.

Enabling factors included distance to the health facility (categorised as a big problem or not a big problem based on TDHS classification) and mother’s autonomy (categorised as involved in household decisions or not involved in household decisions). Need factors included ever contraceptive use (categorised as yes or no) and desire for pregnancy, categorized as desired the pregnancy or no desire for pregnancy. All study factors were categorised based on evidence from past studies from LMICs [16, 32–34, 36, 40, 41, 50–55].

### Statistical analysis

The study analytical strategy was similar to previously published studies [16, 32]. First, initial analyses involved the estimation of frequencies and percentages for each study factor in describing the characteristics of study participants. Second, the prevalence of ‘one to three’ and ‘four or more’ ANC visits was calculated for predisposing, enabling, need and community-level factors for each survey year to examine the extent to which the prevalence decreased or increased over the study period (1999–2016). Third, we estimated *P* for the trend in models of the combined data to determine changes in the outcomes (i.e. 1–3 ANC visits and four or more ANC visits) within each study factor over the study period. Fourth, univariate logistic regression analyses were conducted to investigate the study factors associated with the study outcomes. Fifth, a four-stage multivariate multinomial logistic regression models were then used to examine the association between the predisposing, enabling, need and community-level factors with the outcome variables. The multivariate modelling was based on the adapted Anderson model [42] and employed in previous research [16, 32, 34, 37, 52, 56–59].

Specifically, community-level factors were entered into the stage 1 model to assess their relationship with the outcome variables, with adjustment for predisposing, enabling and need factors. A similar strategy was used in models of predisposing (sociodemographic and maternal media exposure) factors to examine their relationship with the outcome variables, with additional adjustment for enabling, need and community-level factors (stage 2). The same modelling approach was used for the enabling and need factors in the third and fourth stages (stage 3 and 4), respectively. Multivariate models were conducted separately for the 2015–2016 TDHS and the combined dataset to assess any variations in the factors associated with ANC service utilisation in Tanzania. This was done because the most recent nationally representative data potentially reflect the current sociodemographic, economic and health service system context of Tanzania [17, 18].

Furthermore, the TDHS data from 1999 to 2016 were combined in the study for the following reasons: (i) to investigate trends and factors associated with the utilisation of ANC during the MDG era to provide relevant ANC service use data to policymakers and health practitioners during the implementation of the SDG–3.1 agenda, (ii) to provide a unique opportunity for comparison of ANC service use over time and (iii) to improve the statistical power of the study. In models of the combined dataset, an additional adjustment was done for the year of the survey and population weight.

Odds ratios with 95% confidence intervals (CIs) were estimated as the measure of association between the study factors and outcome variables. All statistical analyses were conducted in Stata version 14.0 (Stata Corp, College Station, TX, USA), with ‘svy’ command used to adjust for sampling weights, clustering and stratification; ‘lincom’ command for estimating percentage points changes; and ‘mlogit’ function was used for the multinomial models.

## Results

### Characteristics of the study participants

Over the study period (1999–2016), approximately four out of five (77.9%) of the participants resided in rural areas, and more than one third (75.5%) of women attained at least primary level of education. Nearly half of the women resided in poor households (46.7%), and more than two third (69%) of women listened to the radio. Sixty-two percent of women reported that distance from the health facility was not a big problem, and 56% of the participants were involved in household decision-making (Table 1).

**Table 1:** Characteristics of the study participants

Variables	1999 (***N*** = 3282)	2004–2005 (***N*** = 8725)	2010 (***N*** = 8176)	2015–2016 (***N*** = 10,052)	1999–2016 (***N*** = 30,235)
	*n (%)	*n (%)	*n (%)	*n (%)	*n (%)
**Community-level factors**					
Place of residence					
Urban	614 (18.7)	1691 (19.4)	1660 (20.3)	2727 (27.1)	6692 (22.1)
Rural	2668 (81.3)	7034 (80.6)	6516 (79.7)	7325 (72.9)	23,543 (77.9)
**Predisposing factors**					
**Socio-demographic factors**					
Maternal age					
15–24 years	1123 (34.2)	2819 (32.3)	2488 (30.4)	3134 (31.2)	9564 (31.6)
25–34 years	1501 (45.7)	4231 (48.5)	3680 (45.0)	4426 (44.0)	13,838 (45.8)
35–49 years	659 (20.1)	1674 (19.2)	2008 (24.6)	2492 (24.8)	6833 (22.6)
Maternal education					
No schooling	907 (27.6)	2318 (26.6)	2090 (25.6)	2103 (20.9)	7419 (24.5)
Primary school or higher	2375 (72.4)	6407 (73.4)	6085 (74.4)	7948 (79.1)	22,814 (75.5)
Household wealth status					
Poor	1651 (53.4)	3680 (44.3)	3917 (50.1)	2337 (42.0)	11,585 (46.7)
Middle	1097 (35.5)	3328 (40.1)	2736 (35.0)	2090 (37.5)	9252 (37.3)
Rich	341 (11.0)	1295 (15.6)	1171 (15.0)	1143 (20.5)	3950 (16.0)
Maternal employment					
No employment	591 (49.2)	825 (44.2)	873 (36.1)	1602 (39.0)	3890 (40.5)
Formal employment	101 (8.4)	254 (13.6)	300 (12.4)	641 (15.5)	1297 (13.5)
Informal employment	510 (42.2)	788 (42.2)	1246 (51.5)	1885 (45.7)	4428 (46.0)
**Exposure to media**					
Listening to radio					
No	1452 (44.3)	2485 (28.5)	2754 (33.7)	2576 (25.6)	9267 (30.7)
Yes	1827 (55.7)	6231 (71.5)	5419 (66.3)	7476 (74.4)	20,953 (69.3)
Reading newspapers/magazines					
No	1205 (58.8)	5970 (68.5)	5741 (70.3)	6415 (63.8)	19,331 (66.7)
Yes	844 (41.2)	2752 (31.5)	2422 (29.7)	3635 (36.2)	9652 (33.3)
Watching television					
No	2786 (85.0)	7003 (80.4)	6133 (75.0)	5834 (58.0)	21,756 (72.0)
Yes	496 (15.0)	1713 (19.6)	2043 (25.0)	4218 (42.0)	8470 (28.0)
**Enabling factors**					
Distance to health facilities**					
Big problem		3603 (41.3)	1883 (23.1)	4715 (47.0)	10,201 (38.0)
Not a big problem		5115 (58.7)	6266 (76.9)	5337 (53.0)	16,718 (62.0)
Mother’s autonomy in household**					
Involved in decisions		4219 (48.4)	4479 (54.8)	6407 (63.7)	15,105 (56.0)
Not involved in decisions		4506 (51.6)	3697 (45.2)	3645 (36.3)	11,848 (44.0)
**Need factors**					
Ever contraceptive use					
No	2324 (70.8)	6374 (73.1)	5391 (66.0)	6214 (61.8)	20,303 (67.2)
Yes	958 (29.2)	2350 (26.9)	2785 (34.0)	3838 (38.2)	9931 (32.8)
Desire to the current pregnancy					
Desired the pregnancy	2918 (89.0)	8278 (95.0)	7831 (96.4)	9633 (95.8)	28,660 (95.0)
Not desired the pregnancy	361 (11.0)	438 (5.0)	295 (3.6)	419 (4.2)	1513 (5.0)

*n (%) weighted count and proportion for each outcome variable by study factors

**Variables not reported in the 1999 Tanzania Demographic and Health Survey (TDHS)

Between 1999 and 2016, women who resided in urban households increased from 18.7 to 27.1%, and women who attended primary school or higher increased from 72.4 to 79.1%. Women who resided in rich households increased from 11.0% in 1999 to 20.5% in 2016, while women who had no employment decreased from 49.2% in 1999 to 39.0% in 2016 (Table 1).

### Prevalence of one to three and four or more ANC service use by study factors

In the combined data, the highest proportion of women who made between one and three ANC visits was observed among those who had no schooling (49.6%), followed by those who resided in poor households (48.7%). The lowest proportion of 1–3 ANC service attendance was among women who resided in rich households (29.5%) (Table 2). In the same data, the highest prevalence of four and more ANC service use was in women who were formally employed and those from the rich households (69.0% for each variable), followed by those who resided in urban areas (66.0%). The lowest prevalence of four or more ANC visits was among women who had no schooling (45.8%) (Table 3).

**Table 2:** Prevalence of one to three antenatal care visits by the study factors in Tanzania, 1999–2016

Variables	1999 (***N*** = 568)	2004–2005 (***N*** = 2028)	2010 (***N*** = 3314)	2015–2016 (***N*** = 3028)	1999–2016 (***N*** = 8,938)	% change (95% CI), 1999–2016
	*n (%)	*n (%)	*n (%)	*n (%)	*n (%)	
**Community-level factors**						
Place of residence						
Urban	68 (13.6)	328 (25.8)	556 (44.0)	724 (34.4)	1677 (32.6)	20.7 (14.6, 26.9)
Rural	499 (30.3)	1700 (38.0)	2473 (58.5)	2589 (52.5)	7262 (47.5)	22.1 (17.4, 26.9)
**Predisposing factors**						
**Socio-demographic factors**						
Maternal age						
15–24 years	215 (27.5)	701 (36.3)	972 (56.8)	1092 (47.5)	2981 (44.3)	20.0 (14.6, 25.5)
25–34 years	223 (24.0)	915 (34.8)	1266 (53.2)	1380 (46.4)	3785 (42.4)	22.4 (16.8, 28.0)
35–49 years	129 (29.8)	412 (34.8)	791 (56.5)	841 (47.5)	2173 (45.4)	17.7 (10.9, 24.5)
Maternal education						
No schooling	182 (33.0)	584 (40.1)	797 (61.2)	744 (55.5)	2307 (49.6)	22.4 (14.5, 30.3)
Primary school or higher	385 (24.2)	1445 (33.7)	2231 (53.3)	2569 (45.1)	6631 (42.0)	20.9 (16.7, 25.1)
Household wealth status						
Poor	320 (39.0)	907 (38.8)	1465 (60.6)	1654 (65.2)	3551 (48.7)	26.2 (20.4, 32.0)
Middle	179 (25.0)	780 (36.8)	1063 (56.2)	692 41.5)	2659 (42.4)	16.5 (9.9, 23.2)
Rich	31 (11.6)	242 (24.5)	393 (42.3)	968 (27.3)	917 (29.5)	15.7 (9.5, 21.9)
Maternal employment						
No employment	90 (22.7)	164 (27.4)	317 (49.0)	456 (40.5)	1026 (37.1)	17.9 (7.2, 28.5)
Formal employment	15 (20.5)	40 (20.0)	76 (30.7)	169 (35.0)	299 (29.8)	14.5 (3.4, 25.6)
Informal employment	66 (18.4)	167 (28.1)	418 (45.4)	579 (39.2)	1230 (36.7)	20.8 (13.9, 27.7)
**Exposure to media**						
Listening to radio						
No	271 (30.4)	604 (39.3)	1021 (58.7)	887 (52.7)	2782 (47.6)	22.2 (16.6, 27.9)
Yes	296 (23.6)	1420 (33.8)	2005 (53.5)	2427 (45.3)	6148 (42.2)	21.7 (16.5, 26.8)
Reading newspapers/magazines						
No	206 (27.6)	1469 (39.0)	2157 (57.7)	2207 (51.0)	6039 (48.0)	23.3 (18.1, 28.6)
Yes	100 (15.7)	556 (28.2)	871 (50.0)	1106 (40.8)	2634 (37.3)	25.1 (20.1, 30.1)
Watching television						
No	513 (29.0)	1688 (38.0)	2351 (59.3)	2040 (52.5)	6591 (46.8)	23.5 (18.9, 28.1)
Yes	55 (14.6)	339 (26.3)	678 (44.5)	1274 (40.4)	2345 (37.0)	25.8 (19.8, 31.8)
**Enabling factors**						
Distance to health facilities**						
Big problem		901 (39.1)	694 (57.7)	1631 (50.7)	3227 (48.0)	
Not a big problem		1126 (32.7)	2325 (54.5)	1682 (44.0)	5134 (44.5)	
Mother’s autonomy in household**						
Involved in decisions		971 (34.2)	1643 (52.2)	2047 (44.2)	4662 (43.9)	
Not involved in decisions		1057 (36.4)	1385 (59.0)	1266 (52.4)	3708 (48.4)	
**Need factors**						
Contraceptive use						
No	427 (28.6)	1587 (38.8)	2020 (58.5)	2121 (51.1)	6154 (46.7)	22.4 (17.8, 27.1)
Yes	141 (21.5)	441 (26.6)	1009 (49.5)	1193 (41.3)	2784 (38.4)	19.8 (13.9, 25.7)
Desire to the current pregnancy						
Desired the pregnancy	484 (25.8)	1901 (35.2)	2884 (55.3)	3133 (47.0)	8402 (43.8)	21.1 (16.8, 25.4)
Not desired the pregnancy	83 (31.1)	128 (37.0)	142 (52.7)	180 (49.6)	533 (42.8)	18.5 (8.4, 28.5)

*n (%) weighted counts and the weighted total number vary between categories because of missing values

**Variables not reported in the 1999 Tanzania Demographic and Health Survey (TDHS)

**Table 3:** Prevalence of four or more antenatal care visits by the study factors in Tanzania, 1999–2016

Variables	1999 (***N*** = 1527)	2004–2005 (***N*** = 3547)	2010 (***N*** = 2364)	2015–2016 (***N*** = 3588)	1999–2016 (***N*** = 11,026)	% change (95% CI), 1999–2016
	*n (%)	*n (%)	*n (%)	*n (%)	*n (%)	
**Community-level factors**						
Place of residence						
Urban	430 (85.9)	911 (71.6)	699 (55.2)	1351 (64.1)	3391 (66.0)	− 21.8 (− 28.3, − 15.3)
Rural	1097 (66.6)	2636 (59.0)	1665 (39.4)	2237 (45.3)	7635 (50.0)	− 21.3 (− 27.1, − 15.4)
**Predisposing factors**						
**Socio-demographic factors**						
Maternal age						
15–24 years	551 (70.5)	1180 (61.0)	713 (41.6)	1167 (50.8)	3611 (53.7)	− 19.7 (− 25.8, − 13.7)
25–34 years	689 (74.0)	1638 (62.2)	1065 (44.8)	1537 (51.7)	4929 (55.3)	− 22.3 (− 28.7, − 15.8)
35–49 years	287 (66.1)	730 (61.7)	585 (41.8)	884 (50.0)	2487 (51.9)	− 16.1 (23.8, − 8.4)
Maternal education						
No schooling	330 (59.8)	793 (54.5)	458 (35.1)	552 (41.1)	2133 (45.8)	− 18.7 (− 30.2, − 7.2)
Primary school or higher	1197 (75.0)	2755 (64.2)	1906 (45.5)	3037 (53.3)	8894 (56.4)	− 21.7 (− 26.1, − 17.4)
Household wealth status						
Poor	667 (65.0)	1346 (57.5)	893 (37.0)	591 (39.4)	3497 (48.0)	− 25.5 (− 32.9, − 18.1)
Middle	529 (73.6)	1282 (60.5)	798 (42.2)	889 (57.6)	3498 (55.8)	− 15.9 (− 22.9, − 8.9)
Rich	238 (88.4)	728 (73.6)	529 (57.0)	646 (70.3)	2141 (69.0)	− 18.1 (− 24.3, − 11.8)
Maternal employment						
No employment	291 (73.4)	394 (66.0)	309 (47.7)	642 (57.1)	1636 (59.1)	− 16.3 (− 32.8, − 0.1)
Formal employment	59 (79.5)	157 (78.1)	170 (69.0)	307 (63.6)	692 (69.0)	− 15.9 (− 26.9, − 4.8)
Informal employment	292 (81.6)	405 (68.1)	496 (53.8)	870 (58.8)	2062 (61.5)	− 22.7 (− 29.6, − 15.8)
**Exposure to media**						
Listening to radio						
No	582 (65.2)	864 (56.2)	668 (38.4)	735 (43.6)	2848 (48.7)	− 21.6 (− 29.3, − 13.9)
Yes	943 (75.2)	2683 (63.8)	1696 (45.2)	2854 (53.2)	8176 (56.1)	− 22.0 (− 27.4, − 16.6)
Reading newspapers/magazines						
No	534 (71.5)	2171 (57.5)	1498 (40.1)	2011 (46.4)	6214 (49.3)	− 25.0 (− 30.6, − 19.5)
Yes	535 (84.0)	1377 (70.0)	857 (49.1)	1578 (58.2)	4347 (61.5)	− 25.8 (− 30.8, − 20.8)
Watching Television						
No	1207 (68.2)	2618 (58.8)	1537 (38.8)	1745 (45.0)	7108 (50.5)	− 23.2 (− 28.8, − 17.7)
Yes	320 (85.0)	924 (71.7)	826 (54.2)	1844 (58.4)	3914 (61.7)	− 26.6 (− 32.7, − 20.5)
**Enabling factors**						
Distance to health facilities**						
Big problem		1319 (57.3)	459 (38.2)	1509 (47.0)	3288 (48.9)	
Not a big problem		2225 (64.6)	1895 (44.4)	2079 (54.4)	6200 (53.7)	
Mother’s autonomy in household**						
Involved in decisions		1806 (63.5)	1453 (46.2)	2490 (53.8)	5749 (54.1)	
Not involved in decisions		1741 (60.0)	911 (38.8)	1098 (45.5)	3750 (48.9)	
**Need factors**						
Ever contraceptive use						
No	1018 (68.3)	2375 (58.0)	1362 (39.5)	1931 (46.5)	6686 (50.7)	− 21.8 (− 27.6, − 16.0)
Yes	509 (77.4)	1172 (70.8)	1002 (49.1)	1658 (57.3)	4340 (60.0)	− 20.1 (− 26.2, − 13.9)
Desire to the current pregnancy						
Desired the pregnancy	1352 (72.0)	3349 (62.0)	2240 (43.0)	3421 (51.2)	10362 (54.0)	− 20.7 (− 25.9, − 15.5)
Not desired the pregnancy	175 (65.3)	198 (57.4)	122 (45.2)	167 (46.0)	663 (53.1)	− 19.3 (− 29.5, − 9.1)

*n (%) weighted counts and the weighted total number vary between categories because of missing values

**Variables not reported in the 1999 Tanzania Demographic and Health Survey (TDHS)

### Trends in ANC service use in Tanzania from 1999 to 2016

**Fig. 2:**
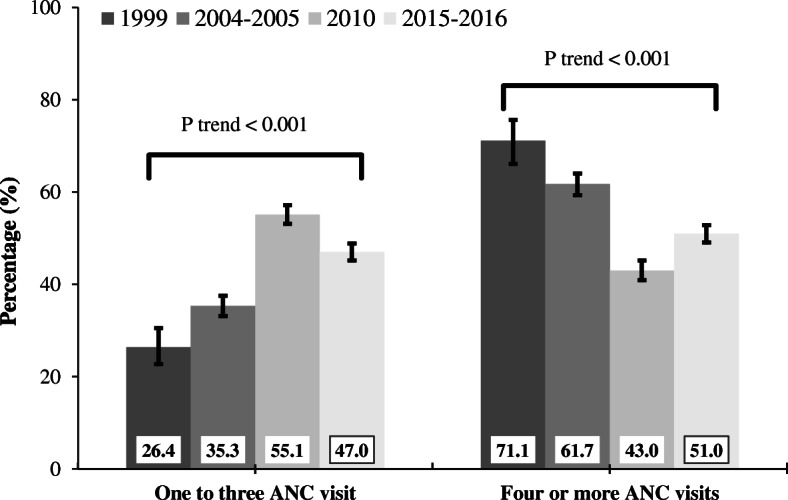
Trends in antenatal care service use in Tanzania, 1999 to 2016. Error bars indicate 95% confidence interval

Between 1999 and 2016, the proportion of women who made one to three ANC visits increased from 26.4% (95% confidence interval [CI] 24.5%, 36.7%) in 1999 to 47.0% (95% CI 45.2%, 48.9%) in 2016 (*P* value < 0.001). In contrast, the proportion of women who made four or more ANC visits decreased from 71.1% (95% CI 65.2%, 82.9%) in 1999 to 51.0% (95% CI 49.1%, 52.8%) in 2016 (*P* value < 0.001) (Fig. 2). Between 1999 to 2016, the highest percentage point change in the proportion of one to three ANC visits was found in poor-level households (% change = 26.2; 95% CI 20.4, 32.0) (Table 2). In the same period, the highest decrease in the proportion of four or more ANC visits was observed among mothers who watched television (% change = − 26.6; 95% CI − 32.7, − 20.5) (Table 3).

### Factors associated with one to three ANC service visits

The odds of attending between one and three ANC visits were significantly higher among women who attained at least primary level of education compared to those who had no schooling (odds ratio (OR) 1.89; 95% CI 1.17, 3.07). Informally employed women were more likely to attend between one and three ANC visits compared to those who were not in employment (OR 1.77; 95% CI 1.13, 2.77). The likelihood of making one to three ANC visits was significantly higher in women who belonged to middle or rich households compared to those who resided in poor households (OR 2.50; 95% CI 1.54, 4.07, for middle households and OR 1.97; 95% CI 1.06, 3.68, for rich households). Women who were not involved in household decision-making were less likely to make between one and three ANC visits compared to their counterparts (OR 0.58; 95% CI 0.35, 0.95) (Table 4).

**Table 4:** Factors associated with 1–3 antenatal care visits in Tanzania, 1999–2016

Variables	1999	2004–2005	2010	2015–2016	1999–2016	***P*** for trend
	OR (95% CI)^**$**^	OR (95 % CI)^**$**^	OR (95 % CI)^**$**^	OR (95 % CI)^**$**^	OR (95 % CI)^**$**^	
**Community-level factors**						
Place of residence						
Urban	1.00	1.00	1.00	1.00	1.00	0.019
Rural	0.95 (0.11–8.46)	0.68 (0.30–1.53)	0.46 (0.14–1.49)	1.16 (0.47–2.85)	0.79 (0.48–1.33)	0.000
**Predisposing factors**						
**Socio-demographic factors**						
Maternal age						
15–24 years	1.00	1.00	1.00	1.00	1.00	0.003
25–34 years	1.20 (0.19–7.57)	0.52 (0.25–1.07)	0.69 (0.25–1.93)	0.80 (0.35–1.83)	0.69 (0.43–1.11)	0.001
35–49 years	2.93 (0.24–35.67)	0.82 (0.32–2.07)	0.95 (0.28–3.15)	1.20 (0.41–3.51)	1.14 (0.63–2.06)	0.004
Maternal education						
No schooling	1.00	1.00	1.00	1.00	1.00	0.000
Primary school and above	6.38 (0.98–41.68)	2.75 (1.31–5.77)	2.33 (0.81–6.70)	0.59 (0.22–1.58)	1.89 (1.17–3.07)	0.000
Household wealth status						
Poor	1.00	1.00	1.00	1.00	1.00	0.000
Middle	2.22 (0.27–18.18)	2.74 (1.31–5.73)	0.82 (0.29–2.27)	4.40 (1.60–12.16)	2.50 (1.54–4.07)	0.013
Rich	2.10 (0.11–38.51)	4.03 (1.60–10.11)	1.32 (0.31–5.72)	1.23 (0.35–4.31)	1.97 (1.06–3.68)	0.058
Maternal employment						
No employment	1.00	1.00	1.00	1.00	1.00	0.000
Formal employment		1.75 (0.37–8.21)	2.27 (0.27–18.98)	1.32 (0.39–4.49)	1.69 (0.73–3.93)	0.480
Informal employment	1.75 (0.27–11.21)	1.48 (0.73–3.01)	2.15 (0.78–5.96)	1.34 (0.61–2.95)	1.77 (1.13–2.77)	0.001
**Exposure to media**						
Listening to radio						
No	1.00	1.00	1.00	1.00	1.00	0.002
Yes	2.01(0.22–18.46)	1.17 (0.56–2.44)	2.61 (0.96–7.10)	2.13 (0.95–4.80)	1.45 (0.92–2.29)	0.000
Reading newspapers/magazines						
No	1.00	1.00	1.00	1.00	1.00	0.000
Yes		0.75 (0.34–1.66)	0.77 (0.25–2.38)	1.13 (0.49–2.65)	0.84 (0.50–1.39)	0.010
Watching television						
No	1.00	1.07 (0.43–2.67)	1.00	1.00	1.00	0.000
Yes	0.14 (0.02–1.12)		0.35 (0.10–1.19)	1.48 (0.55–4.03)	1.08 (0.63–1.85)	0.096
**Enabling factors**						
Distance to the health facilities**						
Big problem		1.00	1.00	1.00	1.00	0.014
Not a big problem		1.54 (0.62–3.79)	1.42 (0.28–7.37)	1.02 (0.49–2.11)	0.92 (0.59–1.45)	0.000
Mother’s autonomy **						
Involved in household decisions		1.00	1.00	1.00	1.00	0.306
Not involved in household decisions		0.27 (0.24–0.31)	1.19 (0.49–2.94)	1.29 (0.58–2.86)	0.58 (0.35–0.95)	0.000
**Need factors**						
Ever contraceptive use						
No	1.00	1.00	1.00	1.00	1.00	0.000
Yes		0.59 (0.28–1.24)	0.86 (0.32–2.34)	0.62 (0.29–1.34)	0.77 (0.49–1.21)	0.006
Desire to the current pregnancy						
Desired	1.00	1.00	1.00	1.00	1.00	0.000
Not desired		0.69 (0.25–1.90)	0.44 (0.09–2.13)	0.70 (0.15–3.29)	0.52 (0.25–1.06)	0.306

^$^Adjusted for community, socio-demographics, health, enabling and need factors as described in the “Methods” section

**Variables not reported in the 1999 Tanzania Demographic and Health Survey (TDHS)

### Factors associated with four or more ANC service visits

The odds of making four or more ANC visits were significantly higher in mothers who attained at least primary level of education compared to those with no schooling (OR 2.25; 95% CI 1.39, 3.65). The likelihood of making four or more ANC visits was significantly higher among mothers who were from middle and rich households compared to mothers who were from poor households (OR 2.73; 95% CI 1.67, 4.44, for middle households and OR 3.14; 95% CI 1.69, 5.53, for rich households). Informally employed women had higher odds of attending four or more ANC visits compared to those who had no employment (OR 1.84; 95% CI 1.18, 2.87). Women who listened to the radio were more likely to receive four or more ANC visits compared to those who did not listen to the radio (OR 1.68; 95% CI 1.06, 2.65). Women who had no desire to be pregnant were less likely to make four or more ANC visits compared to the mothers who desired to be pregnant (OR 0.38; 95% CI 0.19, 0.79) (Table 5).

**Table 5:** Factors associated with four or more antenatal care (ANC) visits in Tanzania, 1999–2016

Variables	1999	2005	2010	2015–2016	1999–2016	***P*** for trend
	OR (95% CI)^**$**^	OR (95% CI)^**$**^	OR (95% CI)^**$**^	OR (95% CI)^**$**^	OR (95% CI)^**$**^	
**Community-level factors**						
Place of residence						
Urban	1.00	1.00	1.00	1.00	1.00	0.313
Rural	0.61 (0.69–5.29)	0.70 (0.31–1.56)	0.57 (0.17–1.84)	0.93 (0.38–2.27)	0.77 (0.47–1.29)	0.010
**Predisposing factors**						
**Socio-demographic factors**						
Maternal age						
15–24 years	1.00	1.00	1.00	1.00	1.00	0.064
25–34 years	1.47 (0.24–9.10)	0.52 (0.25–1.07)	0.73 (0.26–2.04)	0.87 (0.38–1.99)	0.73 (0.45–1.16)	0.100
35–49 years	2.97 (0.25–35.55)	0.89 (0.36–2.23)	0.86 (0.26–2.85)	1.15 (0.39–3.36)	1.05 (0.58–1.89)	0.085
Maternal education						
No schooling	1.00	1.00	1.00	1.00	1.00	0.019
Primary school and above	9.48 (1.49–60.45)	3.20 (1.55–6.61)	2.83 (0.98–8.19)	0.78 (0.29–2.10)	2.25 (1.39–3.65)	0.079
Household wealth status						
Poor	1.00	1.00	1.00	1.00	1.00	0.001
Middle	1.72 (0.21–13.73)	2.93 (1.42–6.04)	0.79 (0.28–2.21)	5.84 (2.11–16.13)	2.73 (1.67–4.44)	0.232
Rich	2.76 (0.16–48.72)	6.52 (2.65–16.07)	1.84 (0.43–7.94)	2.19 (0.63–7.65)	3.14 (1.69–5.83)	0.628
Maternal employment						
No employment	1.00	1.00	1.00	1.00	1.00	0.107
Formal employment	1.67 (0.54–3.87)	2.58 (0.56–11.82)	3.51 (0.42–29.18)	1.38 (0.41–4.66)	2.07 (0.90–4.76)	0.911
Informal employment	2.01 (0.98–4.59)	1.67 (0.83–3.35)	2.61 (0.94–7.23)	1.36 (0.62–2.97)	1.84 (1.18–2.87)	0.039
**Exposure to media**						
Listening to radio						
No	1.00	1.00	1.00	1.00	1.00	0.101
Yes	3.17 (0.35–28.40)	1.13 (0.55–2.34)	2.73 (0.99–7.45)	2.44 (1.09–5.49)	1.68 (1.06–2.65)	0.015
Reading newspapers/magazines						
No	1.00	1.00	1.00	1.00	1.00	0.010
Yes	0.75 (0.54–1.76)	0.79 (0.36–1.73)	0.84 (0.27–2.58)	1.28 (0.55–2.98)	0.97 (0.58–1.61)	0.254
Watching television						
No	1.00	1.00	1.00	1.00	1.00	0.002
Yes	0.17 (0.02–1.32)	1.57 (0.64–3.84)	0.47 (0.14–1.62)	1.68 (0.62–4.57)	1.34 (0.78–2.29)	0.737
**Enabling factors**						
Distance to the health facilities**						
Big problem		1.00	1.00	1.00	1.00	0.214
Not a big problem		1.17 (0.49–2.80)	1.51 (0.29–7.94)	1.08 (0.52–2.25)	0.96 (0.61–1.51)	0.008
Mother’s autonomy **						
Involved in household decisions		1.00	1.00	1.00	1.00	0.989
Not involved in household decisions		0.32 (0.29–1.46)	1.39 (0.57–3.44)	1.13 (0.51–2.50)	0.75 (0.46–1.24)	0.003
**Need factors**						
Ever contraceptive use						
No	1.00	1.00	1.00	1.00	1.00	0.021
Yes	0.54 (0.42–2.14)	0.84 (0.40–1.75)	1.16 (0.43–3.14)	0.81 (0.38–1.76)	0.99 (0.63–1.55)	0.163
Desire to the current pregnancy						
Desired	1.00	1.00	1.00	1.00	1.00	0.006
Not desired	0.64 (0.51–0.86)	0.45 (0.17–1.22)	0.35 (0.73–1.72)	0.40 (0.08–1.90)	0.38 (0.19–0.79)	0.658

^$^Adjusted for community, socio-demographics, health, enabling and need factors as described in the methods section

**Variables not reported in the 1999 Tanzania Demographic and Health Survey (TDHS)

## Discussion

Our study indicates that the proportion of Tanzanian women who made one to three ANC visit increased from 26% in 1999 to 47% in 2016. However, the percentage of women who made four or more ANC visits decreased from 71% in 1999 to 51% in 2016. Women who attained at least primary education, those who resided in wealthier households and were informally employed had higher likelihood of attending one to three ANC visits. Women not involved in household decision-making were associated with a lower odds of attending between one and three ANC visits. Higher maternal education and household wealth, maternal informal employment status and listening to the radio were associated with four or more ANC service visits. Women who did not have a desire for pregnancy were associated with a lower likelihood of attending four or more ANC visits.

Evidence has shown that improved educational status has a vital impact on maternal health service utilization [32, 34, 44, 60, 61]. In Tanzania, reports have indicated that maternal literacy rate has increased over time from 67% in 2004 to 77% in 2016 [15, 25–27], and the improvements in maternal education was also demonstrated in our analyses. The present study showed that women who attained at least primary education were more likely to attend between one and three ANC visits, and this association was even stronger for those who made four or more ANC visits. This finding is similar to studies conducted in Ethiopia [34], Ghana [62, 63], Timor-Leste [64] and Kenya [65], which found that higher maternal education was associated with increased uptake of ANC services. Probable reasons for why educated women have increased ANC use might be that educated women have the potential to engage more with health promotion messages or they may have better insights into the benefits of attending ANC [62]. Educated women are also likely to have the financial resources to pay for direct and indirect costs related to ANC services in a country like Tanzania, where health services are largely provided through out-of-pocket costs [66]. Another possible explanation for this finding is that higher maternal education may increase a woman’s autonomy and household decision-making power. These factors can, in turn, increase the opportunities for women to access quality health care services, including ANC [67]. Our finding provides support for universal primary education in Tanzania to be implemented in line with SDG-4 that aims to improve quality education for all girls and boys by 2030 [68].

The present study indicated that women who resided in wealthier households had an increased likelihood of attending between one and three ANC visits in Tanzania. This association was stronger among women who made four or more ANC visits. Our finding was consistent with studies from many LMICs, which showed that higher household wealth status was related to ANC service utilization [52, 69–73]. Women from wealthier households may have better access to material resources (such as money, cars, or motorcycles) that can facilitate access to ANC services [74]. Additionally, it has been suggested that women who reside in wealthier households may have better access to standard health care facilities (such as hospitals or clinics), and this may have played a role in Tanzania [74]. This is important because of the increase in the proportion of women who resided in rich households between 1999 and 2016.

Past studies conducted in LMICs Nigeria [54], Vietnam [75], Bangladesh [76], India [77, 78] and Cambodia [79] have suggested that maternal employment was associated with ANC service utilization. The current study showed similar associations between maternal employment with one to three and four or more ANC visits in Tanzania. Possible reasons for these associations may be similar to the facilitating factors of women with higher educational attainment and those from wealthier households, where access to essential material resources can facilitate ANC service use of mothers [71]. Addressing socio-economic inequalities and increasing job opportunities for women remain key priority areas to improve the uptake of ANC in Tanzania.

Our study indicated that mothers who were not involved in household decision-making were less likely to attend between one and three ANC visits in Tanzania. The findings were supported by studies conducted in 31 sub-Saharan African countries which indicated that women who were not involved in household decision-making were less likely to fully utilize ANC services [59]. Also, studies conducted in Nepal [80, 81], Bangladesh [82], Senegal [59] and India [83] showed that mothers who did not involve in household decision-making were associated with a reduced likelihood of appropriate ANC service use. Possible reasons for the association between women who did not involve in household decision-making and reduced ANC visits may be that women who have no autonomy in household decision-making anticipated to have a low level of autonomy in health-seeking behaviour, including seeking health care for ANC services. The situation may be worse if there are wide differences in socio-economic status between fathers, and the presence of cultural norms that do not support women within their households and communities may negatively affect health-seeking behaviours of pregnant women [84]. Tackling negative sociocultural norms (such as fear of disclosing pregnancy, seeking permission from village elders and traditional birth attendants to visit health facilities and spousal fidelity) [85] and economic and health service barriers to ANC is significant to increase the health-seeking behaviour of Tanzanian women during pregnancy.

Research conducted in Ethiopia [34], Kenya [86], Benin [51] and India [32] suggested that maternal media exposure (including listening to the radio) was associated with ANC service use. Our study showed that women who listened to the radio had a higher likelihood of attending at least four ANC visits in Tanzania. Listening to the radio may have resulted in frequent ANC use as radio broadcasts may have provided relevant health promotion messages, including the benefits of ANC, as well as information on the danger signs of potential complications associated with pregnancy [34]. Given that the majority of Tanzanian women reside in rural areas and may not have access to visible media tools such as television or print media [15], the dissemination of maternal health promotion messages through the radio may help to increase the uptake of ANC services in Tanzania.

The present study suggested that women who did not desire pregnancy were less likely to make four or more ANC visits compared to those who desired pregnancy. This finding is consistent with research conducted in Nigeria [87], Ethiopia [34] and Tanzania [16], where the desire for pregnancy was related to increased ANC visits. The association between the desire for pregnancy and ANC service use indicates the importance of a woman having a planned pregnancy through the use of family planning methods [34]. Women with unplanned pregnancy have low expectations for the pregnancy, and this can make the woman not to seek skilled ANC services [88]. Additionally, women with unintended pregnancy go through a series of denial stages even after starting ANC visits with a view that the pregnancy will disappear, and they also tend to hide the pregnancy from friends and families [89]. Future studies that specifically consider the impact of a woman’s desire for pregnancy (in the context of family planning) on ANC service utilization may be warranted for continued advocacy to improve maternal health during pregnancy in Tanzania.

## Limitations and strengths

The study has the following limitations. First, the study was based on cross-sectional data which makes the assessment of temporal relationships between the study factors and ANC visits difficult. Nevertheless, the results of our study are consistent with previously published longitudinal studies conducted in LMICs [75, 90, 91]. Second, the ANC data collected in the four TDHS were based on self-reported information which could be a source of recall bias. This may have resulted in misclassification bias in both outcome variables and can subsequently lead to either an over- or under-estimation of the effect size. Third, there was a lack of assessment of all relevant confounders (such as data on health care access, the health status of pregnant women and mother’s psychosocial factors) as this may have provided further information about factors associated with ANC service use in Tanzania. Finally, our study was based on the Measure DHS dataset and the WHO recommendation of four or more ANC visits; however, the WHO has recently recommended that eight or more ANC visits by pregnant women would be more beneficial, particularly in reducing perinatal mortality [11]. The availability of new data on the recent WHO recommendation would have provided additional information on ANC use in Tanzania. Nevertheless, the present study provides valuable insights into where and how national and subnational governments and agencies in Tanzania can begin to make inroads into scaling up maternal health services to meet the new WHO recommendation.

The study also has strengths. The large representative sample, with a high response rate, indicates that selection bias is unlikely to affect the observed results. The use of trained personnel with validated questionnaires in the TDHS is also likely to reduce measurement bias in the study. Finally, the study provides valuable insights into key factors associated with ANC visits in Tanzania and potentially an opportunity for policymakers and public health practitioners to design and implement focused maternal health interventions to improve ANC service use in Tanzania.

## Conclusion

Our study shows that one to three ANC attendance increased during the MDG era in Tanzania. However, four or more ANC attendance decreased over the same period. Women who attained at least primary education, those who were in wealthier households and those who were informally employed had a higher likelihood to attend between one to three ANC visits. Four or more ANC service attendance was associated with higher maternal education and household wealth, maternal informal employment and listening to the radio. Among Tanzanian women, increasing ANC service attendance is achievable if national and potentially sub-national health and social policies and programmes focus on key modifiable maternal factors of education, household wealth and employment.

## Data Availability

Information on the data and content can be accessed at https://dhsprogram.com/data/available-datasets.cfm

## Abbreviations

ANC: Antenatal care visit
AOR: Adjusted odds ratio
BOT: Bank of Tanzania
CI: Confidence interval
DHS: Demographic and Health Survey
EAs: Enumeration areas
HIV/AIDS: Human immunodeficiency virus/acquired immunodeficiency disease syndrome
ICF: Inner City Fund
LMICs: Low-middle-income countries
UNICEF: The United Nations Children’s Fund
NIMR: National Institute for Medical Research
MDGs: Millennium Development Goals
OCGS: Office of the Chief Government Statistician
SDGs: Sustainable development goals
SVY: Survey
TDHS: Tanzania Demographic and Health Survey
USAID: United State of America Aid
WHO: World Health Organization
MoHCDEC: Ministry of Health Community Development Elderly and Children
MoFEA: Ministry of Finance and Economic Affairs
MoCLA: Ministry of Constitution and Legal Affairs

## References

[1] World Health Organization. Maternal mortality: factsheet Online: World Health Organization; 2016 [cited 2019 June 16]. Available from: http://www.who.int/mediacentre/factsheets/fs348/en/.

[2] Alvarez JLG, Ruth Hernández, alentín Gil, Angel. Factors associated with maternal mortality in Sub-Saharan Africa: an ecological study. BMC public health. 2009;9(1):462. 10.1186/1471-2458-9-462 PMC2801510 20003411

[3] World Health Organization. Trends in maternal mortality: 1990-2015: estimates from WHO, UNICEF, UNFPA, World Bank Group and the United Nations Population Division: executive summary. . 2015.

[4] Kinney MV, Kerber KJ, Black RE, Cohen B, Nkrumah F, Coovadia H (2010). Sub-Saharan Africa’s mothers, newborns, and children: where and why do they die?. PLoS medicine..

[5] Ronsmans C, Graham WJ (2006). Maternal mortality: who, when, where, and why. The lancet Maternal Survival Series steering group..

[6] Say LC (2014). Doris Gemmill, Alison Tunçalp, Özge Moller, Ann-Beth Daniels, Jane Gülmezoglu, A Metin Temmerman, Marleen Alkema, Leontine. Global causes of maternal death: a WHO systematic analysis. The Lancet Global Health..

[7] Alkema LC (2016). Doris Hogan, Daniel Zhang, Sanqian Moller, Ann-Beth, Gemmill AF, Doris Ma Boerma, Ties, Temmerman MM, Colin. Global, regional, and national levels and trends in maternal mortality between 1990 and 2015, with scenario-based projections to 2030: a systematic analysis by the UN Maternal Mortality Estimation Inter-Agency Group. The Lancet..

[8] WHO. Trends in maternal mortality: 1990-2015: estimates from WHO, UNICEF, UNFPA, World Bank Group and the United Nations Population Division: executive summary. World Health Organization, 2015.

[9] Lincetto OM-A, Seipati Gomez, Patricia Munjanja, Stephen. Practical data, policy programmatic support for newborn care in Africa. Opportunities for Africa's newborns. 2006:55-62.

[10] World Health Organization (2002). WHO antenatal care randomized trial: manual for the implementation of the new model.

[11] World Health Organization. WHO recommendations on antenatal care for a positive pregnancy experience: World Health Organization. 2016. 28079998

[12] UNICEF. Antenatal care 2018.

[13] UNICEF W, World bank, UN-DESA Population Divison. Levels & trends in child mortality report 2013 estimates developed by the UN Inter-agency Group for child mortality estimation. 2013.

[14] Ogbo FA, Ezeh OK, Awosemo AO, Ifegwu IK, Tan L, Jessa E (2019). Determinants of trends in neonatal, post-neonatal, infant, child and under-five mortalities in Tanzania from 2004 to 2016. BMC Public Health..

[15] United Republic of Tanzania. Tanzania demographic and health survey and malaria indicator survey 2015-2016 final report,. 2016.

[16] Gupta S, Yamada G, Mpembeni R, Frumence G, Callaghan-Koru JA, Stevenson R (2014). Factors associated with four or more antenatal care visits and its decline among pregnant women in Tanzania between 1999 and 2010. PloS one..

[17] Allison S. Tanzania’s president targets corruption with surprise visits and sackings Johannesburg: Global Development; 2015 [cited 2015 8th December]. Available from: https://www.theguardian.com/global-development/2015/dec/08/tanzania-new-president-john-magufuli-targets-corruption-surprise-visits-sackings.

[18] Paget D. Magufuli has been president for two years: how he’s changing Tanzania Online: The Conversation; 2017 [cited 2019 October 2]. Available from: http://theconversation.com/magufuli-has-been-president-for-two-years-how-hes-changing-tanzania-86777.

[19] Gross KS, Joanna Armstrong Kessy, Flora Pfeiffer, Constanze Obrist, Brigit. Timing of antenatal care for adolescent and adult pregnant women in south-eastern Tanzania. BMC Pregnancy and Childbirth. 2012;12(1):16. 10.1186/1471-2393-12-16 PMC3384460 22436344

[20] Gross KS, Joanna Armstrong Kessy, Flora Pfeiffer, Constanze Obrist, Brigit. Antenatal care in practice: an exploratory study in antenatal care clinics in the Kilombero Valley, south-eastern Tanzania. BMC pregnancy and childbirth. 2011;11(1):36. 10.1186/1471-2393-11-36 PMC3123249 21599900

[21] Mrisho MO, Brigit Schellenberg, Joanna Armstrong Haws, Rachel A Mushi, Adiel K, Mshinda HT, Marcel Schellenberg, David. The use of antenatal and postnatal care: perspectives and experiences of women and health care providers in rural southern Tanzania. BMC Pregnancy and childbirth. 2009;9(1):10. 10.1186/1471-2393-9-10 PMC2664785 19261181

[22] Nyamtema AS, Bartsch-de Jong A, Urassa DP, Hagen JP, van Roosmalen J (2012). The quality of antenatal care in rural Tanzania: what is behind the number of visits?. BMC pregnancy and childbirth..

[23] SDG-UN. Transforming our world: The 2030 agenda for sustainable development. New York, USA: UN; 2015. Available from: https://sustainabledevelopment.un.org/content/documents/21252030%20Agenda%20for%20Sustainable%20Development%20web.pdf.

[24] United Nations. Sustainable development - knowledge platform 2017 [cited 2019 13 October]. Available from: : https://sustainabledevelopment.un.org/sdg3.

[25] United Republic of Tanzania. Tanzania reproductive and child health survey 1999. 1999.

[26] United Republic of Tanzania. Tanzania demographic and health survey 2004-2005. 2005.

[27] United Republic of Tanzania. Tanzania demographic and health survey 2010. 2010.

[28] Croft, Trevor N., Aileen M. J. Marshall, Courtney K. Allen, et al. Guide to demographic and health survey Statistics. Rockville, Maryland, USA: ICF; 2018.

[29] Bureau National of Statistics. The 2012 population and housing census: basic demographic and socio-economic profile Dar es Salaam, Tanzania. 2014.

[30] Bureau National of Statistics. Population census 1988. National profile, Dar es Salaa Tanzania. 1988.

[31] Agho KE, Ezeh OK, Ogbo FA, Enoma AI, Raynes-Greenow C. Factors associated with inadequate receipt of components and use of antenatal care services in Nigeria: a population-based study. International health. 2018;10(3):172-81. doi: 10.1093/inthealth/ihy011 %J International Health. 10.1093/inthealth/ihy011 29562242

[32] Ogbo FA, Dhami MV, Ude EM, Senanayake P, Osuagwu UL, Awosemo AO (2019). Enablers and barriers to the utilization of antenatal care services in India. International journal of environmental research and public health..

[33] Ogbo FA, Ogeleka P, Awosemo AO (2018). Trends and determinants of complementary feeding practices in Tanzania, 2004–2016. Tropical medicine and health..

[34] Mekonnen TD (2019). Tinashe Perz, Janette Ogbo, Felix Akpojene Trends and determinants of antenatal care service use in Ethiopia between 2000 and 2016. International journal of environmental research public health..

[35] Tsegay Y, Gebrehiwot T, Goicolea I, Edin K, Lemma H, San SM (2013). Determinants of antenatal and delivery care utilization in Tigray region, Ethiopia: a cross-sectional study. International journal for equity in health..

[36] Simkhada BT, Edwin R (2008). van Porter, Maureen Simkhada, Padam. Factors affecting the utilization of antenatal care in developing countries: systematic review of the literature. Journal of advanced nursing..

[37] De Allegri M, Ridde V, Louis VR, Sarker M, Tiendrebéogo J, Yé M, et al. Determinants of utilisation of maternal care services after the reduction of user fees: a case study from rural Burkina Faso. Health Policy. 2011;99(3):210-8. doi: 10.1016/j.healthpol.2010.10.010. PubMed PMID: 104817419. Language: English. Entry Date: 20110624. Revision Date: 20150711. Publication Type: Journal Article. . 10.1016/j.healthpol.2010.10.010 21056505

[38] Dairo M, Owoyokun K. Factors affecting the utilization of antenatal care services in Ibadan, Nigeria. Benin Journal of Postgraduate Medicine. 2010;12(1).

[39] Adamu Y, Salihu H (2002). Barriers to the use of antenatal and obstetric care services in rural Kano, Nigeria. Journal of obstetrics and gynaecology..

[40] Rai RKS (2013). Prashant Kumar Kumar, Chandan Singh, Lucky. Factors associated with the utilization of maternal health care services among adolescent women in Malawi. Home health care services quarterly..

[41] Guliani HS (2013). Ardeshir Serieux, John. Determinants of prenatal care use: evidence from 32 low-income countries across Asia, Sub-Saharan Africa and Latin America. Health Policy and Planning..

[42] Andersen RN, John F. Societal and individual determinants of medical care utilization in the United States. The Milbank Memorial Fund Quarterly Health and Society. 1973:95–124. 10.1111/j.1468-0009.2005.00428.x. 4198894

[43] Babalola SF, Adesegun. Determinants of use of maternal health services in Nigeria - looking beyond individual and household factors. BMC Pregnancy and Childbirth. 2009;9(1):43. doi: 10.1186/1471-2393-9-43. 10.1186/1471-2393-9-43 PMC2754433 19754941

[44] Efendi F, Chen C-M, Kurniati A, Berliana SM (2017). Determinants of utilization of antenatal care services among adolescent girls and young women in Indonesia. Women & health..

[45] Neupane SD, Teye D (2012). Determinants of time of start of prenatal care and number of prenatal care visits during pregnancy among Nepalese women. Journal of community health..

[46] Vyas S, Kumaranayake L (2006). Constructing socio-economic status indices: how to use principal components analysis. Health Policy and Planning..

[47] Lakew Y, Tabar L, Haile D (2015). Socio-medical determinants of timely breastfeeding initiation in Ethiopia: evidence from the 2011 nationwide demographic and health survey. Int Breastfeed J..

[48] Ogbo FA, Page A, Idoko J, Claudio F, Agho KE (2015). Trends in complementary feeding indicators in Nigeria, 2003–2013. BMJ Open..

[49] Ahmed KY, Page A, Arora A, Ogbo FA. Trends and factors associated with complementary feeding practices in Ethiopia from 2005 to 2016.n/a(n/a):e12926. doi: 10.1111/mcn.12926. 10.1111/mcn.12926 PMC7083482 31833239

[50] Aliyu AA, Dahiru T. Predictors of delayed Antenatal Care (ANC) visits in Nigeria: secondary analysis of 2013 Nigeria Demographic and Health Survey (NDHS). The Pan African medical journal. 2017;26. doi: 10.11604%2Fpamj.2017.26.124.9861. 10.11604/pamj.2017.26.124.9861 PMC5429423 28533847

[51] Dansou J, Adekunle AO, Arowojolu AO (2017). Factors associated with antenatal care services utilisation patterns amongst reproductive age women in Benin Republic: an analysis of 2011/2012 benin republic’s demographic and health survey data. Nigerian Postgraduate Medical Journal..

[52] Nketiah-Amponsah ES (2013). Bernardin Arthur, Eric. Determinants of utilization of antenatal care services in developing countries: recent evidence from Ghana. African Journal of Economic Management Studies.

[53] Tewdros BGm, A. Dibaba, Y. Factors affecting antenatal care utilization in Yem Special District, South western Ethiopia. Ethiopian Journal of health sciences. 2009;19.

[54] Onah HEI, Lawrence C (2006). Iloabachie, Gabriel C. Factors associated with the use of maternity services in Enugu, southeastern Nigeria. Social science & medicine..

[55] Gebremeskel FD, Yohannes Admassu, Bitiya. Timing of first antenatal care attendance and associated factors among pregnant women in Arba Minch Town and Arba Minch District, Gamo Gofa Zone, South Ethiopia. Journal of environmental and public health. 2015;2015. doi: 10.1155/2015/971506. 10.1155/2015/971506 PMC4620253 26543485

[56] Babalola SF, Adesegun. Determinants of use of maternal health services in Nigeria-looking beyond individual and household factors. BMC pregnancy and childbirth. 2009;9(1):43. 10.1186/1471-2393-9-43 PMC2754433 19754941

[57] Dahiru T, Oche OM. Determinants of antenatal care, institutional delivery and postnatal care services utilization in Nigeria. Pan African medical journal. 2015;21(1). doi: 10.11604/pamj.2015.21.321.6527. 10.11604/pamj.2015.21.321.6527 PMC4633744 26587168

[58] Magadi MA, Madise NJ, Rodrigues RN (2000). Frequency and timing of antenatal care in Kenya: explaining the variations between women of different communities. Social Science & Medicine..

[59] Chol C, Negin J, Agho KE, Cumming RG (2019). Women’s autonomy and utilisation of maternal healthcare services in 31 Sub-Saharan African countries: results from the demographic and health surveys, 2010–2016. BMJ open..

[60] Mekonnen YM (2003). Asnakech. Factors influencing the use of maternal healthcare services in Ethiopia. Journal of Health, Population and utrition..

[61] Abegaz KH, Habtewold EM (2019). Trend and barriers of antenatal care utilization from 2000 to 2016 Ethiopian DHS: a data mining approach. Scientific African..

[62] Dickson KSD, Eugene Kofuor Maafo Kumi-Kyereme, Akwas, Ahinkorah BO. Determinants of choice of skilled antenatal care service providers in Ghana: analysis of demographic and health survey. Maternal health, neonatology and perinatology. 2018;4(1):14. 10.1186/s40748-018-0082-4 PMC6040073 30002866

[63] Ganle JKP (2014). Michael Fitzpatrick, Raymond Otupiri, Easmon Inequities in accessibility to and utilisation of maternal health services in Ghana after user-fee exemption: a descriptive study. International journal for equity in health..

[64] Khanal VC (2015). Jonia Lourenca Nunes Brites Mishra, Shiva Raj Karkee, Rajendra Lee, Andy H Under-utilization of antenatal care services in Timor-Leste: results from Demographic and Health Survey 2009–2010. BMC pregnancy childbirth.

[65] Wang WA (2011). Soumya Wang.

[66] Kibusi SM, Sunguya BF, Kimunai E, Hines CS (2018). Health insurance is important in improving maternal health service utilization in Tanzania—analysis of the 2011/2012 Tanzania HIV/AIDS and malaria indicator survey. BMC health services research..

[67] Chanana K (1996). Educational attainment status production and womens autonomy: a study of two generations of Punjabi women in New Delhi.

[68] Abel GJ, Barakat B, Kc S, Lutz W (2016). Meeting the Sustainable Development Goals leads to lower world population growth. Proceedings of the National Academy of Sciences..

[69] Tsawe MS (2019). A Sathiya.

[70] Doku D, Neupane S, Doku PN (2012). Factors associated with reproductive health care utilization among Ghanaian women. BMC International Health and Human Rights..

[71] Arthur E (2012). Wealth and antenatal care use: implications for maternal health care utilisation in Ghana. Health economics review..

[72] Alam NH, Mohammad, Dumont AF, Pierre. Inequalities in maternal health care utilization in sub-Saharan African countries: a multiyear and multi-country analysis. PloS one. 2015;10(4):e0120922. doi: 10.1371/journal.pone.0120922. 10.1371/journal.pone.0120922 PMC4390337 25853423

[73] Ahmed SC, Andreea A. Gillespie, Duff G. Tsui, Amy O. Economic status, education and empowerment: implications for maternal health service utilization in developing countries. PLOS ONE. 2010;5(6):e11190. doi: 10.1371/journal.pone.0011190. 10.1371/journal.pone.0011190 PMC2890410 20585646

[74] Ahmed S, Creanga AA, Gillespie DG, Tsui AO (2010). Economic status, education and empowerment: implications for maternal health service utilization in developing countries. PloS one..

[75] Ha BT, Tac PV, Duc DM, Duong DT, Thi LM (2015). Factors associated with four or more antenatal care services among pregnant women: a cross-sectional survey in eight South Central Coast provinces of Vietnam. International journal of women’s health..

[76] Kamal SMH, Che Hashim Islam, Md Nazrul. Factors associated with the timing of antenatal care seeking in Bangladesh. Asia Pacific Journal of Public Health. 2015;27(2):NP1467-NP80. doi: 10.1177%2F1010539513485786. 10.1177/1010539513485786 24097925

[77] Pallikadavath S, Foss M, Stones RW (2004). Antenatal care: provision and inequality in rural north India. Social Science & Medicine..

[78] Jat RTN, N. Sebastian, M. Factors affecting the use of maternal health services in Madhya Pradesh state of India: a multilevel analysis. Int J Equity Health. 2011;10. doi: 10.1186/1475-9276-10-59. 10.1186/1475-9276-10-59 PMC3283453 22142036

[79] Wang WH, Rathavuth. Levels and determinants of continuum of care for maternal and newborn health in Cambodia-evidence from a population-based survey. BMC pregnancy childbirth. 2015;15(1):62. 10.1186/s12884-015-0497-0 PMC4371879 25885596

[80] Adhikari R (2016). Effect of women’s autonomy on maternal health service utilization in Nepal: a cross sectional study. BMC women's health..

[81] Thapa DKN (2013). Anke. Women’s autonomy and husbands’ involvement in maternal health care in Nepal. Social Science & Medicine..

[82] Rahman MM, Mostofa MG, Hoque MA (2014). Women’s household decision-making autonomy and contraceptive behavior among Bangladeshi women. Sexual & Reproductive Healthcare..

[83] Mistry R, Galal O, Lu M (2009). Women’s autonomy and pregnancy care in rural India: a contextual analysis. Social science & medicine..

[84] Lowe M, Chen D-R, Huang S-L (2016). Social and cultural factors affecting maternal health in rural Gambia: an exploratory qualitative study. PloS one..

[85] Roberts J, Hopp Marshak H, Sealy DA, Manda-Taylor L, Mataya R, Gleason P (2017). The role of cultural beliefs in accessing antenatal care in Malawi: a qualitative study. Public Health Nursing..

[86] Achia TN, Mageto LE (2015). Individual and contextual determinants of adequate maternal health care services in Kenya. Women & health..

[87] Aliyu AA, Dahiru T. Predictors of delayed Antenatal Care (ANC) visits in Nigeria: secondary analysis of 2013 Nigeria Demographic and Health Survey (NDHS). The Pan African medical journal. 2017;26:124-. doi: 10.11604/pamj.2017.26.124.9861. PubMed PMID: 28533847. 10.11604/pamj.2017.26.124.9861 PMC5429423 28533847

[88] Abosse Z, Woldie M, Ololo S. Factors influencing antenatal care service utilization in hadiya zone. Ethiopian Journal of Health Sciences. 2010;20(2). 10.4314/ejhs.v20i2.69432 PMC3275839 22434964

[89] Weller RH, Eberstein IW, Bailey M (1987). Pregnancy wantedness and maternal behavior during pregnancy. Demography..

[90] Tekelab TC, Catherine Smith, Roger Loxton, Deborah. Factors affecting utilization of antenatal care in Ethiopia: a systematic review and meta-analysis. PloS one. 2019;14(4):e0214848-e. doi: 10.1371/journal.pone.0214848. PubMed PMID: 30973889. 10.1371/journal.pone.0214848 PMC6459485 30973889

[91] Okedo-Alex IN, Akamike IC, Ezeanosike OB, Uneke CJ (2019). Determinants of antenatal care utilisation in sub-Saharan Africa: a systematic review. BMJ Open..

